# Multi-Platform Omics Analysis Reveals Molecular Signatures for Pathogenesis and Activity of Systemic Lupus Erythematosus

**DOI:** 10.3389/fimmu.2022.833699

**Published:** 2022-04-19

**Authors:** Xiaolan Huang, Laurence Don Wai Luu, Nan Jia, Jia Zhu, Jin Fu, Fei Xiao, Chunyan Liu, Shengnan Li, Gaixiu Shu, Jun Hou, Min Kang, Dan Zhang, Yingjie Xu, Yi Wang, Xiaodai Cui, Jianming Lai, Jieqiong Li, Jun Tai

**Affiliations:** ^1^ Experimental Research Center, Capital Institute of Pediatrics, Beijing, China; ^2^ School of Biotechnology and Biomolecular Sciences, University of New South Wales, Sydney, NSW, Australia; ^3^ Division of Paediatric Rheumatology, Children’s Hospital Affiliated Capital Institute of Paediatrics, Beijing, China; ^4^ Department of Respiratory Disease, Beijing Pediatric Research Institute, Beijing Children’s Hospital, Capital Medical University, National Center for Children’s Health, Beijing, China; ^5^ Department of Otolaryngology, Head and Neck Surgery, Children’s Hospital Affiliated Capital Institute of Pediatrics, Beijing, China

**Keywords:** systemic lupus erythematosus, proteomic, metabolomic, lipidomic, pathogenesis

## Abstract

Systemic lupus erythematosus (SLE) is a complex autoimmune disease with heterogeneous clinical manifestations and the pathogenesis of SLE is still unclear. Various omics results have been reported for SLE, but the molecular hallmarks of SLE, especially in patients with different disease activity, using an integrated multi-omics approach have not been fully investigated. Here, we collected blood samples from 10 healthy controls (HCs) and 40 SLE patients with different clinical activity including inactive (IA), low activity (LA), and high activity (HA). Using an integrative analysis of proteomic, metabolomic and lipidomic profiles, we report the multi-omics landscape for SLE. The molecular changes suggest that both the complement system and the inflammatory response were activated in SLEs and were associated with disease activity. Additionally, activation of the immunoglobulin mediated immune response were observed in the LA stage of the disease, however this immune response was suppressed slightly in the HA stage. Finally, an imbalance in lipid metabolism, especially in sphingolipid metabolism, accompanied with dysregulated apolipoproteins were observed to contribute to the disease activity of SLE. The multi-omics data presented in this study and the characterization of peripheral blood from SLE patients may thus help provide important clues regarding the pathogenesis of SLE.

## Introduction

Systemic lupus erythematosus (SLE), a complex autoimmune disease with heterogeneous clinical manifestations, is characterized by an abnormal immune and inflammatory response (1). Whereas mild symptoms of SLE mainly affect the skin and joints, life-threatening illness involves multiple damage including hematological, neuropsychiatric, and cardiovascular induced by chronic inflammation or the immune system (2). As a consequence, SLE is still difficult to define and treat.

The pathogenesis of SLE is still unclear, but it is thought to be multifactorial involving aberrations in the immune system, as well as heritable, hormonal, and environmental factors, contributing to the tissue damage (1). Systemic tissue damage may arise from a series of complex factors (3), and as a consequence, may alter the proteins and metabolites involved in SLE activity. These proteomic and metabolic disorders indirectly reflect the status of the patients, and may be associated with multiple severe complications (4). Therefore, it will be worthwhile to analyze if host-derived proteins and metabolites in the circulation system are connected to the pathogenesis and activity of SLE.

Recently, the advancement of multi-omics research has aimed to resolve the complexity of the underlying molecular mechanism of diseases (5). Serum is a primary carrier of small molecules; whose relative concentrations directly reflect the physiological status of the organism and the pathogenesis of diseases. Therefore, serum proteins or metabolites are reported to be able to distinguish SLE from healthy controls (HCs). For example, a label free-based two dimensional liquid chromatography mass spectrometry platform (2D-LC-MS/MS) was used to identify potential biomarkers for SLE diagnosis (6). The metabolite profiles of SLE has also been previously investigated by several research groups and they showed that glycolysis and mitochondrial oxidative metabolism are activated in SLE patients. Additionally, lipid metabolism was also reported to be significantly altered in SLE patients and this change was correlated with disease activity (7). However, SLE is a multistep process with different stages of disease. Currently, the protein or metabolite profile which make up each distinct stage-specific phenotype has not yet been elucidated, hindering complete understanding of the pathogenesis of SLE. The combined roles of multi-omics in the pathogenesis of SLE has also not been determined. Thus, a comprehensive analysis of the proteome, metabolome and lipidome might therefore aide in uncovering the complexity of SLE.

To gain further insight into the molecular characteristics of SLE patients, a cohort of 10 HCs and 40 SLE patients, classified by clinical activity into inactive (IA), low activity (LA), and high activity (HA), were collected for multi-omics analysis. The primary aim of this study was to determine the proteomic, metabolic and lipidomic signatures of SLEs. The secondary aim was to determine the underlying pathogenies of SLE as well as disease activity. Our multi-omics insight into this disease will contribute to greater understanding of the underlying pathogenesis of SLE.

## Material And Methods

### Patient Enrollment

Blood samples from 40 SLE patients were collected between 26^th^ of December 2019 and 8^th^ of May 2021 from the Capital Institute of Pediatrics, Beijing. The SLE cases were divided into three subgroups according to the SLE Disease Activity Index 2000 (SLEDAI-2k): (i) IA subgroup: SLEDAI ≤4; (ii) LA subgroup: SLEDAI=5-9; and (iii) HA subgroup: SLEDAI ≥10 (8, 9). HCs were enrolled from children who underwent a health checkup at the Capital Institute of Pediatrics. This study was reviewed and approved by the Capital Institute of Pediatrics ethics committee (DWLL2018004). Written informed consent has been signed by patients or their parents.

### Clinical Index

Laboratory findings including C-reactive protein (CRP), erythrocyte sedimentation rate (ESR), serum complement levels (C3, C4), B cell numbers and percentage, IgG, IgA and IgM were detected. Serological screening for SLE included antinuclear antibody (ANA) and anti-double stranded DNA antibodies (anti-dsDNA), anti-smith nuclear antigen (anti-Sm) and anti-nuclear-ribonuclear-protein (anti-nRNP). Pro-inflammatory cytokines including tumor necrosis factor (TNF)-α, interleukin (IL)-2R, IL-10 and IL-6 were measured by chemiluminescence immunoassay technology (CLIA) kit and detected with the Image Immunochemistry System Immulite1000 (Siemens, USA).

### Proteomic Analysis

Firstly, serum samples from each participant were lysed at 25°C for 30 min. The lysates were then reduced with Tris phosphine (Pierce, USA) and incubated at 37°C for 30 min with shaking. Then, iodoacetamide (Sigma-Aldrich, USA) was added for alkylation at 25°C, with agitation at 300 rpm for 1 h without light. Mass spectrometry-grade trypsin gold (Promega, USA) was then added to digest proteins overnight at 37°C. Next, 20μL loading buffer (1% formic acid, FA; 1% acetonitrile, ACN) was used to dissolve the dried peptides. Ten μL of product was then used for liquid chromatography-tandem mass spectrometry (LC-MS/MS) analysis on an Orbitrap Fusion Lumos in data dependent acquisition (DDA) mode coupled with Ultimate 3000 (Thermo Fisher Scientific, USA).

MS detection parameters were set as follows: the ultra-high field Orbitrap analyzer adopts full MS survey scan with a resolution of 120,000 and an ion trap size of 500,000. Mass range was set at 300 to 1400 m/z. MS/MS was carried out in an IonTrap, and the top 20 peptides with the highest energy were identified for fragmentation. Peptides were screened for fragments with 2-7 charges (5×10^3^ AGC target, 35 ms maximum ion time). Maxquant (Version 1.6.17) was used to analyze the mass spectrometry data. Maxquant parameters were set as follows: 20 ppm was the tolerance of precursor ion mass; full cleavage by trypsin was selected; a maximum of two missed cleavages was allowed; cysteine carbamidomethylation was set as a static modification; methionine oxidation and acetylation of peptides’ N-termini were set as variable modifications. Homo sapiens FASTA database (uniprot-proteome UP000005640.fasta) were used for protein searches with following the criteria: (1) peptide length ≥6 amino acids; (2) False Discovery Rate (FDR) ≤1% at the PSM, peptide and protein levels. Peptides were quantified using the peak area derived from their MS1 intensity by Perseus 1.6.15.0; R 3.6. The protein intensity was calculated by the intensity of unique and razor peptides.

### Metabolomic Analysis

For each sample extraction, 400 μL of methanol (MeOH)/ACN (1:1, v/v) solvent mixture was added to 100 μL of serum (2:2:1 ratio, no H_2_O added) and violated vigorously for 5 min, incubated for 1 h at -20°C, and centrifuged for 10 minutes at 13,500 g at 4°C. Then, the supernatant of each sample was transferred to a new tube. To ensure data quality, quality control (QC) samples were collected, containing equal amounts of sera (0.75mL) from the 50 participants, and divided into 4 samples. The QC samples were pretreated in parallel with the study samples and added between each set of runs to monitor the stability. Two platforms, reverse-phase/ultra-performance liquid chromatography (RP/UPLC)-MS/MS methods with positive ion-mode electro spray ionization (ESI) and negative-ion mode ESI, were used to detect the metabolites.

ACQUITY 2D UPLC system (Waters, USA) and TripleTOF 5600+ (AB SCIEX, MA, USA) with ESI source and mass analyzer were applied in all UPLC-MS/MS methods. The mobile solutions used in the gradient elution were water and methanol containing 0.1% FA. The mass spectrometry analysis, with the scan range set at 70-1,500 m/z, alternated between MS and data-dependent MS2 scans using dynamic exclusion. The MS-DIAL software was used for raw data pre-processing, peak alignment and peak annotation. Then, metabolites were identified by matching the retention time, accurate mass, and MS/MS fragmentation data to the MS-DIAL software database and Human Metabolome Database (HMDB, https://hmdb.ca).

### Lipidomic Analysis

Serum samples were mixed with 400uL methyl tert-butyl ether (MTBE) and 80uL MeOH (MTBE : MeOH:H2O=10:2:5, v/v) and vortexed for 30 s. The organic and aqueous phases were separated through centrifugation at 3,000 rpm for 15 min. The upper MTBE was transferred to a new microcentrifuge tube and dried. The dried extracts were then reconstituted with 100 μL of dichloromethane: MeOH (1:1,v/v).

Liquid chromatography (LC) was performed using an ExionLC AD system (AB SCIEX, USA). Phenomenex Kinetex 1.7u EVO C18 columns (2.1×50mm,100A; Agilent, USA) were used for LC separation and a total of 3μL of sample was loaded. The column temperature and flow rate was set to 40°C and 0.5 mL/min, respectively. The mobile phase A contained 50% water, 50% ACN, and 10mM ammonium formate while the mobile phase B contained 10% ACN, 90% isopropyl alcohol (IPA), and 10mM ammonium formate. MS was performed on a 5600+ quadrupole-time of flight/mass spectra (Q-TOF/MS, AB SCIEX, USA). The positive and negative ion ionization mode of electrospray ionization source was used. IDA-TOF-MS/MS with the quadrupole scanning rage set at 50-1500 m/z was used to collect the data. An independent reference, lock-mass ion, *via* the MS-DIAL (ver.3.70; 17 April 2021) was used to ensure mass accuracy during data acquisition. MS-DIAL Lipidomics MSP database was used to identify the assigned modified metabolite ions. To reduce false positive matches, chromatographic retention behavior was considered.

### Statistical Analysis

#### Statistical Analysis of Clinical Data

Clinical data were analyzed using SPSS 16.0 and expressed as mean ± standard deviation (SD). Differences between 3 or 4 groups were calculated using one-way ANOVA while student’s t-test was used to compare between two groups. The level of significance was set at P < 0.05.

#### Statistical Analysis of Multi-Omics Data

We conducted Shapiro test for normality and found that the data from each group fit the normal distribution. For each pair of groups compared, the fold-change (FC) was calculated using the mean from that patient group (e.g. mean of IA vs mean of HC). Two-sided unpaired Welch’s t test was performed for each pair of groups compared. Statistically significant altered proteins, metabolites and lipids were identified using the following criteria: P value < 0.05 and |FC| >1.5 (log_2_FC| >0.58). Partial least squares-discriminate analysis (PLS-DA) was conducted using MetaboAnalyst 4.0 (http://www.metaboanalyst.ca/MetaboAnalyst/). The PLS-DA models were cross-validated using a 10-fold method with unit variance scaling.

Volcano plots were calculated using a combination of FC and t tests, and the intensity data of these regions were used for GraphPad analysis, hierarchical clustering analysis, and metabolic pathway analysis. Heatmaps of the differential proteins, metabolites and lipids were created using the Multi Experiment Viewer software (MeV, version 4.7.4). Metaboanalyst 4.0 web portal and KEGG were used for pathway analysis. Analysis of different sub-clustering models of proteins were performed using Mfuzz v.2.46.0. String, a plug-in for Cytoscape (v.3.2.1), was used to build connected networks of the differentially expressed proteins. Correlation analysis were performed by SPSS16.0.

## Results

### Sample Cohort and Experimental Design

**Figure 1:**
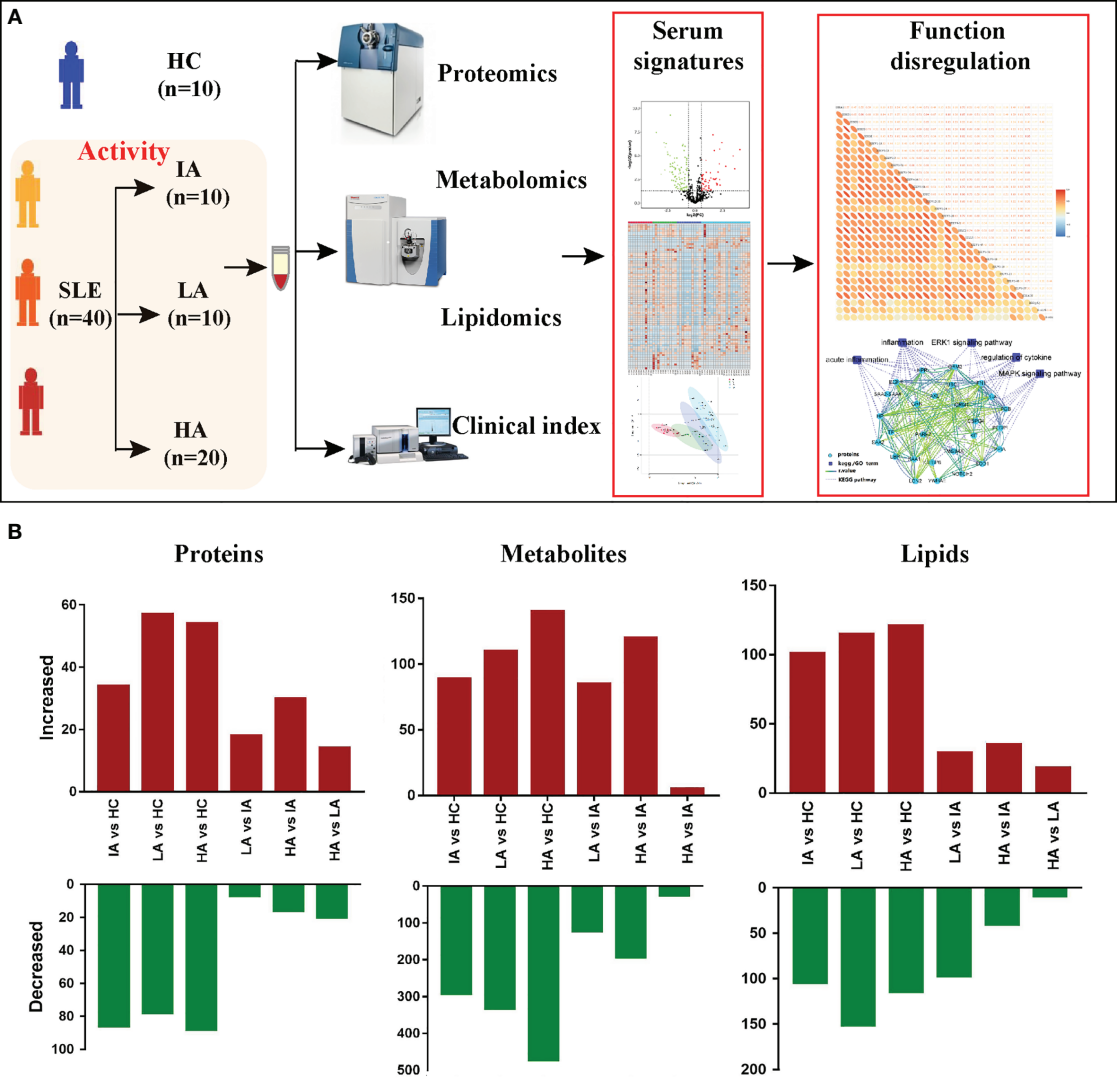
Patients, Study Design, and Multi-omics Profiling of SLEs. **(A)** Study overview. Fifty subjects including 10 HCs and 40 SLEs were recruited for multi-omics profiling analysis and investigation of the underlying pathogenesis. **(B)** Bar plot showing the numbers of significantly differentially expressed proteins, metabolites, and lipids in six pairwise comparisons.

To elucidate the molecular characteristics of SLE patients, 10 HCs and 40 SLE patients with different activity: IAs, LAs and HAs, were collected for this multi-omics study. Throughout the course of this study, we measured autoantibodies, clinical parameters and cytokines. LC-MS/MS was applied to identify the proteomic, metabolomic, and lipidomic signatures in SLE patients. Meanwhile, these omic datasets were then integrated to provide an insight into the underlying pathogenesis of SLE (**Figure 1A**). To quantify the molecular features related to disease activity, we used pairwise comparisons between the four groups for each omic level to identify differentially expressed proteins, metabolites and lipids, respectively **(** **Figure 1B**).

### Clinical and Laboratory Features

The demographic characteristics and laboratory results of enrolled patients are shown in **Table 1**. Increase in disease activity was associated with a gradual increase in disease score. Compared with LAs and HAs, IA patients had a lower percentage of positive anti-dsDNA (P = 0.048), implying a lower disease activity in IAs. Indicators of systemic inflammation, such as ESR (P = 0.0011), and pro-inflammatory cytokines, including TNF-α (P = 0.0127) and IL-10 (P = 0.0186), were increased as disease activity increased. Our observations are consistent with previous findings, which demonstrated that TNF-α and IL-10 levels were significantly increased in active SLE and correlated with clinical SLEDAI-2k (10). In contrast, expression of C3 (P <0.0001) and C4 (P = 0.013) were decreased in SLE patients compared to HCs, showing a decreased trend as disease activity increased.

**Table 1:** Demographic characteristics of the participants.

Variable	HC (n=10)	SLE patients			P value
		IA (n=10)	LA (n=10)	HA (n=20)	
Gender (male/female)	5/5	3/7	1/9	3/17	0.118
Age, mean ± SD	12 ± 0.471	12.52 ± 0.877	10.6 ± 0.521	11.35 ± 0.530	0.2325
Score, mean ± SD	0 ± 0	1.7± 0.597	7.5 ± 0.373	14.7 ± 1.093	<0.0001
Autoantibodies, n (%)					
ANA	/	10 (100)	10 (100)	20 (100)	1.000
Anti-dsDNA	/	3 (30)	7 (70)	15 (75)	0.048
Anti-SM	/	1 (10)	4 (40)	6 (30)	0.304
Anti-nRNP	/	1 (10)	3 (30)	10 (50)	0.089
Complement, mean ± SD					
C3 (g/L)	1.193 ± 0.063	0.995 ± 0.096	0.673 ± 0.075	0.537 ± 0.077	<0.0001
C4 (g/L)	0.194 ± 0.024	0.18 ± 0.032	0.099 ± 0.019	0.090 ± 0.025	0.013
Inflammation markers, mean ± SD					
ESR (mm/60min)	4.9 ± 0.875	15.6 ± 4.812	50.9 ± 12.89	41.1 ± 7.219	0.0011
CRP (mg/L)	0.78 ± 0	2.382 ± 1.108	11.88 ± 7.544	3.1 ± 0.884	0.1173
TNF-α (pg/ml)	5.693 ± 0.412	18.76 ± 6.563	22.21 ± 2.445	25.72 ± 4.002	0.0127
IL-2R (U/ml)	419 ± 54.75	876.3 ± 333.1	1179 ± 209.1	1249 ± 213.7	0.2057
IL-10 (pg/ml)	5 ± 0	7.788 ± 1.834	9.875 ± 3.038	15.08 ± 2.479	0.0186
IL-6 (pg/ml)	2.775 ± 0.349	7.038 ± 3.29	6.554 ± 2.272	8.081 ± 1.836	0.3395
B cells and antibody, mean ± SD					
B cells (no.)	416.6 ± 33.32	175.9 ± 24.71	468.8 ± 107.3	242.4 ± 42.89	0.0033
B cells (%)	17.3 ± 0.473	14.38 ± 1.523	21.21 ± 3.086	14.71 ± 1.909	0.1261
IgG (g/L)	11.64 ± 0.636	16.55 ± 2.567	20.61 ± 3.155	16.97 ± 1.528	0.0441
IgA (g/L)	1.505 ± 0.115	1.25 ± 0.1545	2.136 ± 0.236	2.11 ± 0.258	0.0285
IgM (g/L)	0.975 ± 0.125	2.084 ± 0.389	1.779 ± 0.236	1.541 ± 0.162	0.0189

SLE, systemic lupus erythematosus; HC, healthy control; IA, inactivity; LA, low activity; HA, high activity; ANA, antinuclear antibody; dsDNA, double-stranded DNA; SM, Smith nuclear antigen; nRNP, nuclear-ribonuclear-protein; C3, serum complement 3; C4, serum complement 4; ESR, erythrocyte sedimentation rate; CRP, C-reactive protein; TNF, tumor necrosis factor; IL, interleukin.

Moreover, the number of B cells (P = 0.0033) as well as the levels of antibodies, including IgG (P = 0.0441), IgA (P = 0.0285) and IgM (P = 0.0189) were increased in IAs and LAs, but decreased slightly in HAs. Our observations are consistent with previous findings in SLE where abnormalities in B cells were identified to be involved in the pathogenesis of SLE (11).

### Multi-Omic Profiling for SLE

After data preprocessing, the final dataset contained 3399 analytes, including 777 proteins, 1994 metabolites, and 628 complex lipids that were identified. Next, pairwise comparisons were applied to identify differentially expressed molecules, and identify the molecular signatures of SLE for each omic level.

#### Proteomic Changes in SLE

**Figure 2:**
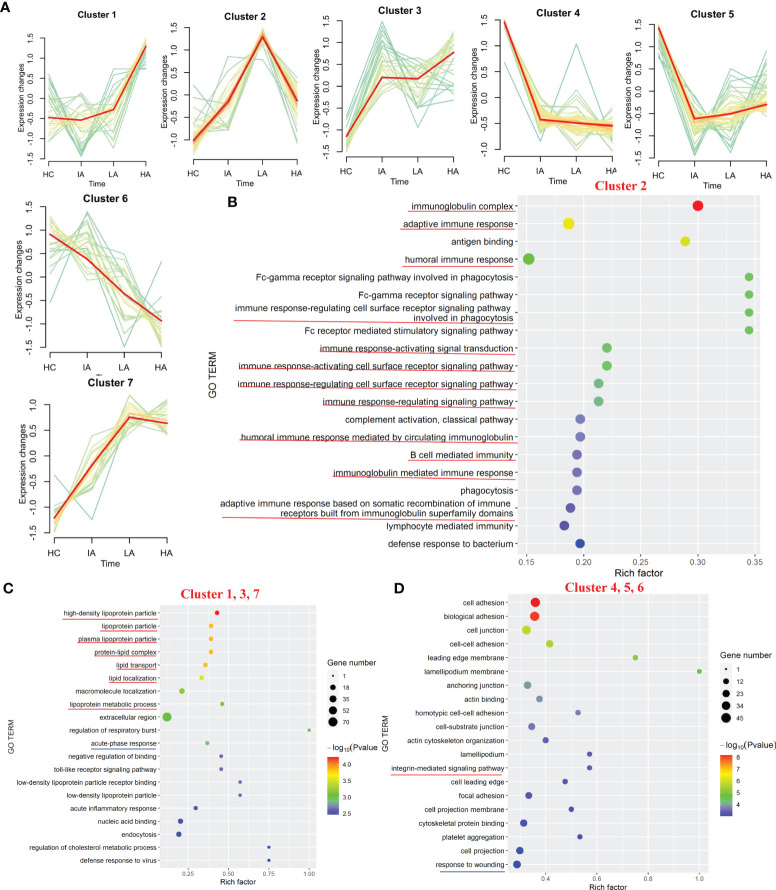
Landscape of Proteins in SLE. **(A)** Hierarchical clustering shows seven differential expression patterns across four groups. **(B)** GO terms enriched in cluster 2. Top 20 GO terms are shown. Red lines highlight immune-related terms. **(C)** GO terms enriched in the increasing clusters (C1, C3 and C7). Top 20 GO terms are shown. Red lines highlight lipoprotein-related terms. Blue line highlights the acute phase response terms. **(D)** GO terms enriched in the decreasing clusters (C4, C5 and C6). Top 20 GO terms are shown. Red line highlights the integrin-mediated signaling pathway term. Blue line highlights the response to wounding term.

Of the 777 proteins (**Supplementary Figure 1A**), 231 were identified as differentially expressed among the four groups (**Supplementary Data 1.1**). We applied the PLS-DA analysis (**Supplementary Figure 1B**) and volcano plots (**Supplementary Figure 1C**) to show the different proteins. Seven expression patterns, including an inverted “V” cluster (C2), three increasing clusters (C1, C3 and C7), and three decreasing clusters (C4, C5 and C6), were identified across patients with different disease activity (**Figure 2A** and **Supplementary Data 2.1**).

Based on the seven expression clustering patterns, a variety of biological pathways were identified to be specifically enriched in patients with different disease severity. As shown in **Figure 2B**, C2 were enriched for proteins involved in immunoglobulin-related immune response pathways (**Supplementary Data 2.2**). Although expression of most immunoglobulin-related proteins were elevated in SLE patients, interestingly, we found that these levels decreased slightly in the HA group compared to LAs. This suggests that immunoglobulin-related immune responses, which were activated in SLEs, might be specifically suppressed in the HAs group. For the three increasing clusters (C1, C3 and C7), these contained proteins primarily involved in lipoprotein-related pathways and acute-phase response pathways (**Figure 2C** and **Supplementary Data 2.2**). The expression levels of apolipoproteins continuously increased in SLEs compared to HCs, suggesting that dysregulation of lipid metabolism in SLEs is associated with disease activity. For proteins in the three decreasing clusters (C4, C5 and C6), these were enriched in integrin-mediated signaling pathways and response to wounding (**Figure 2D**, **Supplementary Figure 2** and **Supplementary Data 2.2**). Proteins found in the three decrease clusters might reflect the increased tissue damage and cell death associated with disease activity (**Figure 2D**).

#### Landscape of Metabolomic Profiles in SLE

**Figure 3:**
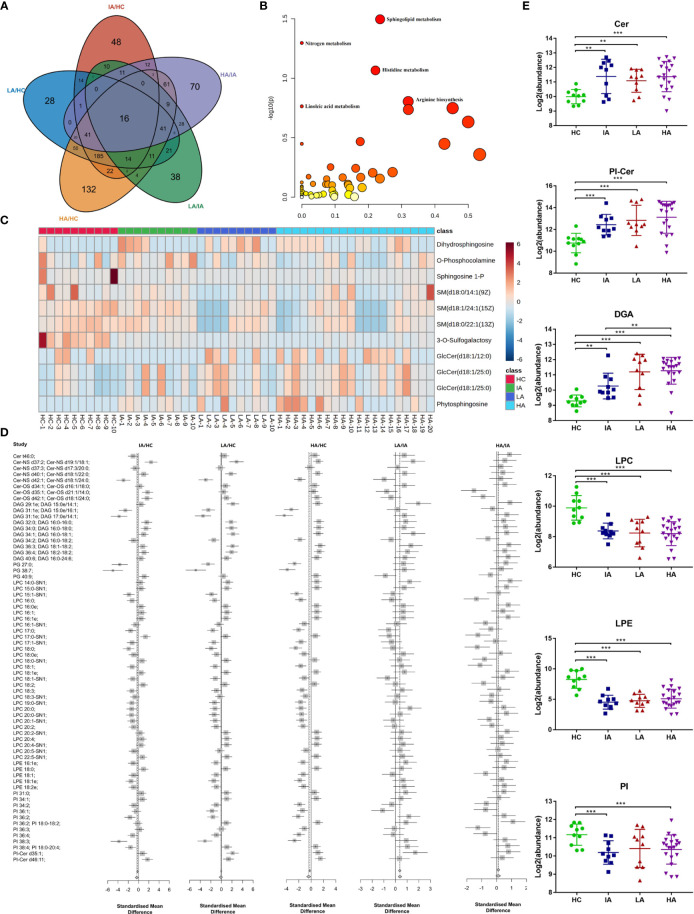
Landscape of Metabolites and Lipids in SLE. **(A)** Venn diagram showing the overlapping number of differentially expressed metabolites in HC, IA, LA, and HA groups. **(B)** Pathway analysis of metabolomics data identified significant effects of SLE on sphingolipid metabolism. **(C)** Heatmap representing expression of metabolites in sphingolipid metabolism in HCs, IAs, LAs and HAs. **(D)** Representative lipid expression changes across four groups. Square and bars represent the mean and standard deviation, respectively. Squares that are left of the line represent ratios <1, while square that are right of the line represent ratios >1. **(E)** Representative lipid expression changes across four groups. Square and bars represent the mean and standard deviation, respectively. P values were calculated using Student’s t test and significant P values are shown in the boxplot. ** means P value < 0.01, and *** means P value < 0.001.

To ensure reliability, a PLS-DA score plot (**Supplementary Figure 3A**) and relative standard deviation (RSD) % of all detected variables (**Supplementary Figure 3B**) were generated. Volcano plots from the metabolomic analyses highlight differentially expressed metabolites identified between each pairwise comparison (**Supplementary Figure 3C**). A summary of the differentially expressed metabolites in each pairwise comparison is shown in **Supplementary Data 1.2**. These differentially expressed metabolites were then used to characterize the metabolic pathways associated with different SLE disease activity (**Figure 3A** **).**

Notably, KEGG analysis indicted that sphingolipid metabolism was the top most significant pathway impacted and associated with SLE and disease activity (**Figure 3B** and **Supplementary Data 3.1**). Interestingly, a previous study suggested that sphingolipid metabolism may be a biomarker and therapeutic target for SLE (12). Consistently, we also observed that the expression of many metabolites in the sphingolipid pathway were gradually changed in SLE patients compared to HCs (**Figure 3C**). This suggests that sphingolipid metabolism may participate in the pathogenesis of SLE and supports the hypothesis that dysregulated lipid metabolism occurs in SLE.

#### Altered Lipid Profiles in SLE

We next investigated the lipid dynamics within the different disease activity groups. A PLS-DA score plot (**Supplementary Figure 4A**) and RSD% of all detected variables (**Supplementary Figure 4B**) were generated. Volcano plots analyses highlight the lipids that were altered between each pairwise comparison (**Supplementary Figure 4C**). These differentially expressed lipids are expressed in **Supplementary Data 1.3**.

Consistent with the metabolomics results above, sphingolipids, including ceramides (Cers), ceramide phosphoinositols (PI-Cers), and most classes of diradylglycerols (DAGs) were significantly increased in SLEs (IAs, LAs and HAs) compared to HCs (**Figures 3D, E**). Of particular interest, the expression of Cers were slightly decreased in LAs and HAs when compared to IAs (**Figure 3E**). Moreover, we observed a reduction in the major classes of serum glycerophospholipids including lysophosphatidylcholine (LPC), glycerophosphoethanolamines (LPE) and glycerophosphoinositols (PI). This indicates an enhanced phospholipase A2 activity in SLE patients compared to HCs (**Figures 3D, E**). LPCs are the major component of the cell membrane and can increase the production of pro-inflammatory cytokines. LPE were previously reported to prime the plant immune system however their function in mammals has not been well characterized (13). Here, down-regulated of LPC and LPE in SLE might imply the immune disorders of HAs (**Figure 3E**). Thus, an analysis of the serum lipidomic signatures, combined with the identification of increased apolipoprotein expression (**Figure 2C**) revealed that lipid changes, especially for sphingolipids is essential for SLE disease activity.

### Dysfunctional Immune and Activated Inflammatory Response in High Activity Stage of SLE

SLE is a multifactorial and complex autoimmune disease, which is characterized by various immune-related and inflammation-related molecular aberrations. Although dysregulated immune responses and inflammation responses in SLE have been reported, the comprehensive analysis and the underling molecules participating the process at different stages of SLE disease activity is limited (14).

**Figure 4:**
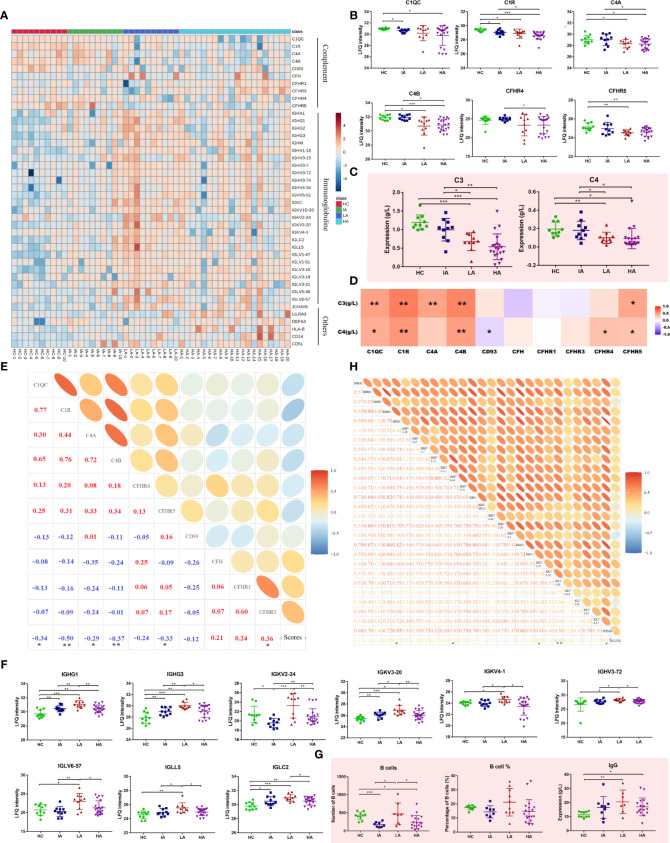
Dysfunctional Immune Response in High Activity Stage of SLE. **(A)** Heatmap depicting the levels of differentially expressed proteins related to immune responses, including complement, immunoglobulins and others in HCs, IAs, LAs, and HAs. **(B)** Expression of representative complement-related proteins across four groups. **(C)** Expression of serum complement C3 and C4 across four groups. Square and bars represent the mean and standard deviation, respectively. P values were calculated using Student’s t test and significant P values are shown. * means P value < 0.05, ** means P value < 0.01, and *** means P value < 0.001. **(D)** Spearman correlation heatmap between levels of differentially expressed proteins and complements. Red, purple, and white denote relatively higher, lower, and mean levels, respectively. * means correlation P value < 0.05. ** means correlation P value < 0.01. **(E)** Correlation analysis of complement-related proteins and SLE disease scores. Red and blue represent positive and negative correlation, respectively. * means correlation P value < 0.05. ** means correlation P value < 0.01. **(F)** Boxplots of representative immunoglobulin-related protein abundances across four groups. **(G)** Number of B cells, percentage of B cells and IgG level across four groups. P values were calculated using Student’s t test and significant P values are shown. * means P value < 0.05, ** means P value < 0.01, and *** means P value < 0.001. **(H)** Correlation analysis of the immunoglobulin-related proteins and SLE disease score. * means correlation P value < 0.05. ** means correlation P value < 0.01.

In this study, many complement components were significantly changed in SLEs, with the trend becoming more pronounced as disease activity increased (**Figure 4A**). As shown in **Figure 4B**, multiple complement-related proteins, including complement C1q subcomponent subunit C (C1QC), complement subcomponent C1r (C1R), complement C4-A (C4A), and complement C4-B (C4B) were downregulated in SLE, especially in LAs and HAs. Besides, the similar expression patterns of serum complements C3 and C4 were observed in SLEs (**Figure 4C**). The levels of C1QC, C1R, C4A, and C4B were positively associated with levels of C3 and C4 (**Figure 4D** and **Supplementary Data 4.1**). More interestingly, they were also negatively related to disease score of SLE (**Figure 4E** and **Supplementary Data 4.2**). This suggests that the complement system was activated in SLE and participated in the activity of SLE. Moreover, many immunoglobulins, such as immunoglobulin heavy constant gamma (IGHG)1, IGHG3, immunoglobulin kappa variable (IGKV) 2-24, and others were significantly increased in SLEs **(** **Figure 4F** **)**. Interestingly, a new pattern of immune disorders at HA stage was observed. Many immunoglobulin proteins, which were increased in LA subgroup of SLEs, were specifically decreased in the HAs **(** **Figure 4F**). Consistently, the number of B cells and levels of IgG also showed similar expression patterns (**Figure 4G**), and were positively correlated with the immunoglobulins levels (**Supplementary Figure 5A** and **Supplementary Data 4.3**). Some immunoglobulin proteins, such as IGHM, IGHV 3-34, IGLV1-47, IGLV 3-10 were positive correlated with disease score of SLE (**Figure 4H** and **Supplementary Data 4.2**). Collectively, changed proteins in HAs implied the activation of complement system and suppression of immunoglobulin-related immune response at high activity stage.

**Figure 5:**
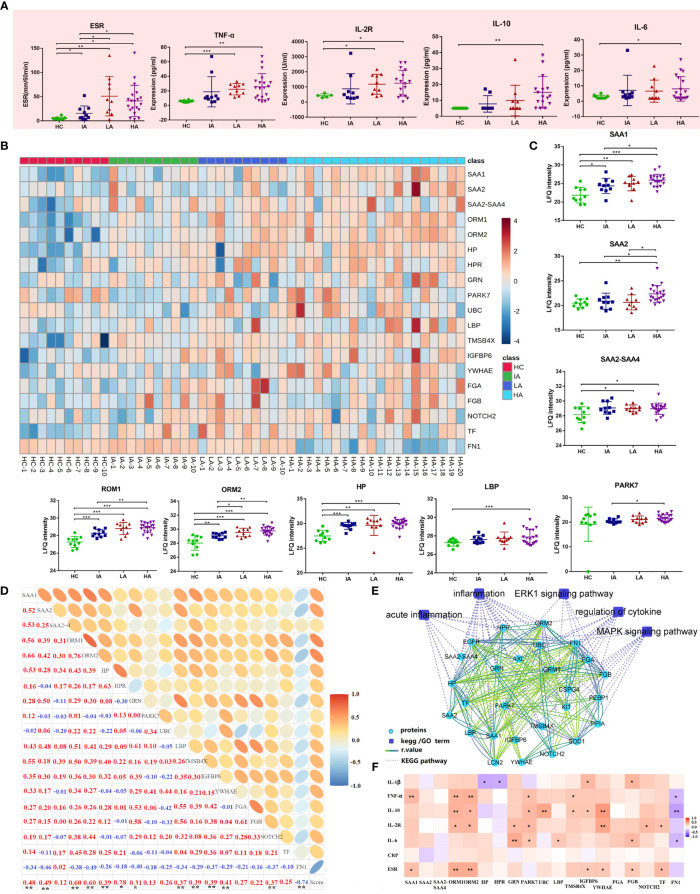
Activated Inflammatory Response in SLE. **(A)** Expression of inflammatory markers and cytokines across four groups. P values were calculated using Student’s t test and significant P values are shown. * means P value < 0.05, ** means P value < 0.01, and *** means P value < 0.001. **(B)** Heatmap depicting expression levels of inflammation-related proteins in HCs, IAs, LAs, and HAs. **(C)** Expression of representative inflammation-related proteins across four groups. P values were calculated using Student’s t test and significant P values are shown. * means P value < 0.05, ** means P value < 0.01, and *** means P value < 0.001. **(D)** Correlation analysis of the inflammatory-related proteins and SLE disease score. Red and blue numbers represent positive and negative correlation, respectively. * means correlation P value < 0.05. ** means correlation P value < 0.01. **(E)** The interaction diagram of proteins involved in acute inflammation, inflammation, ERK1 signaling pathway, regulation of cytokine, MAPK signaling pathway. Blue squares represent the pathways; Green circle represent the changed proteins; Dotted lines represent the association between the pathways and the proteins. Solid lines represent the association between proteins. **(F)** Spearman correlation heat map between levels of differentially expressed proteins and clinical parameters for inflammation. Red, purple, and white denote relatively higher, lower, and mean levels, respectively. * means correlation P value < 0.05. ** means correlation P value < 0.01. *** means P value < 0.001.

Next, we analyzed the expression of inflammation markers in different groups. Levels of ESR and pro-inflammatory cytokines, including TNF-α, IL-2R, IL-6 and IL-10 were elevated in SLE and this was associated with disease activity (**Figure 5A**). Besides, many proteins associated with inflammation identified by proteomics were also up-regulated (**Figure 5B** and **Figure 5C**). For example, the levels of acute-phase proteins (serum amyloid A [SAA] 1, SAA2, SAA2-SAA4) were increased in SLEs (**Figure 5C**). Circulatory SAA concentration was reported to be significantly increased during acute phase responses (15). Here, the expression of SAA1 was higher in SLEs compared to HCs, and was also further elevated in HAs compared to IAs (**Figure 5C**). The level of SAA2 was highest in HAs while the levels of SAA2-SAA4 was higher in LAs and HAs compared to HCs. More interestingly, the levels of SAA1 and SAA2 were positively correlated with SLE disease score (**Figure 5D** and **Supplementary Data 4.2**), and SAA1 alone was correlated with ESR and TNF-α (**Figure 5F** and **Supplementary Data 4.4**). Except for acute inflammatory-related pathway, mitogen-activated protein kinase (MAPK) signaling pathways and extracellular signal-regulated kinase (ERK) 1/2 signaling pathway were also changed in SLE patients (**Figure 5E**).

### Dysregulated Lipid Metabolism in SLE

**Figure 6:**
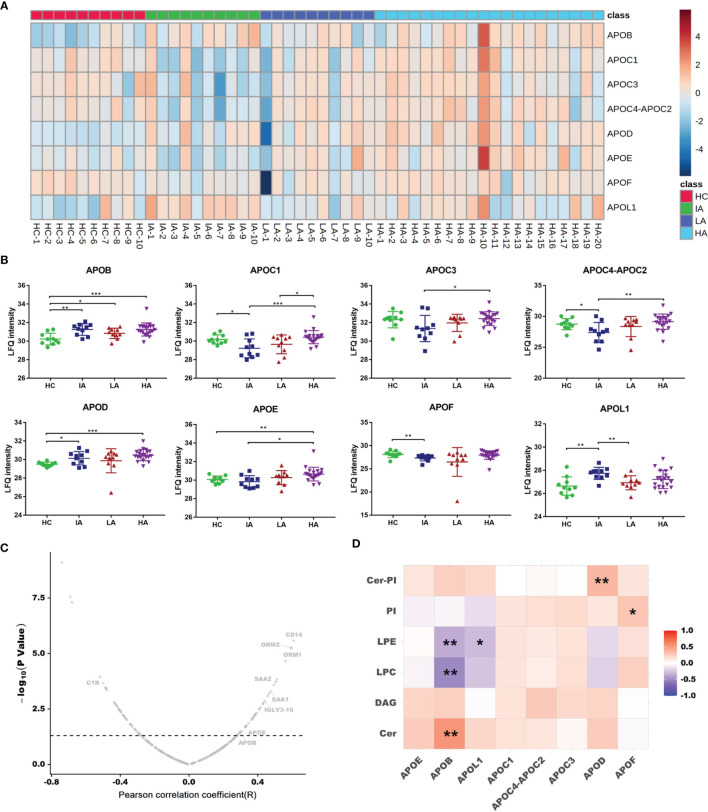
Dysregulated Lipid Metabolism in SLE. **(A)** Heatmap showing expression levels of apolipoproteins in HCs, IAs, LAs, and HAs. **(B)** Abundance levels of apolipoproteins across four groups. P values were calculated using Student’s t test and significant P values are shown. * means P value < 0.05, ** means P value < 0.01, and *** means P value < 0.001. **(C)** Correlation analysis of proteins and SLE disease score. x axis indicates Spearman’s correlation coefficients, and the y axis indicates the significance of the correlation (–log10 of *P* values for each correlate). Dotted line means P value of 0.05. Representative proteins, including APOB and APOE were related to disease score. **(D)** Heatmap of Spearman correlation coefficients between the levels of differentially expressed apolipoproteins and representative lipids. Red, purple, and white denote relatively higher, lower, and mean levels, respectively. * means correlation P value < 0.05. ** means correlation P value < 0.01.

Since chronic inflammation in patients with SLE can lead to accelerated atherosclerosis, it is reasonable to suspect that these risk factors can induce specific alterations in lipoprotein metabolism (16). In this study, we found a large number of apolipoproteins that were up-regulated in SLE, especially in HAs (**Figure 6A**). Compared with HCs, the expressions of apolipoprotein B (APOB), apolipoprotein C (APOC), apolipoprotein D (APOD), apolipoprotein E (APOE) and apolipoprotein L1 (APOL1) were increased in SLE patients (**Figure 6B**). Moreover, the expression of APOB and APOE were correlated to SLE disease score (**Figure 6C**). These results are consistent with previous findings demonstrating that APOE correlated with disease activity and related cytokines in SLE patients (17). Besides, APOC3 was strongly related to cardiovascular disease risk (18). Thus, high levels of APOC3 in HAs may be involved in the occurrence of atherosclerosis induced SLE.

As a lipid transport carrier, apolipoproteins participate in the pathogenesis of SLE, however, their relationship with lipids in SLE is still unclear (17). Here, we conducted a correlation analysis for apolipoproteins and lipid metabolism. As mentioned above, Cer, PI-Cer and DAG were increased, while LPC, LPE and PI were decreased as SLE disease activity increased (**Figure 3E**). Interestingly, we found the expression of APOB were positively connected with Cer, while negatively associated with LPC and LPE (**Figure 6D** and **Supplementary Data 4.5**). Further correlation between apolipoproteins and lipids are provided in **Supplementary Figure 5B**. In addition, many lipids were also observed to be correlated with SLE disease score (**Supplementary Figure 6A**). It has been reported that dyslipidemia occurs frequently in SLE and is characterized by elevated plasma levels of low-density lipoprotein (LDL), APOB, and decreased level of high-density lipoprotein (HDL) (16). Many studies have shown that dyslipidemia, is an important contributing factor that can also accelerate inflammation, autoimmunity, and atherosclerosis in autoimmune diseases (19). Therefore, an imbalance in lipid metabolism together with dysregulated apolipoproteins combined involved in the SLE activity.

### Sphingolipid Metabolism Perturbed in High Activity Stage of SLE

**Figure 7:**
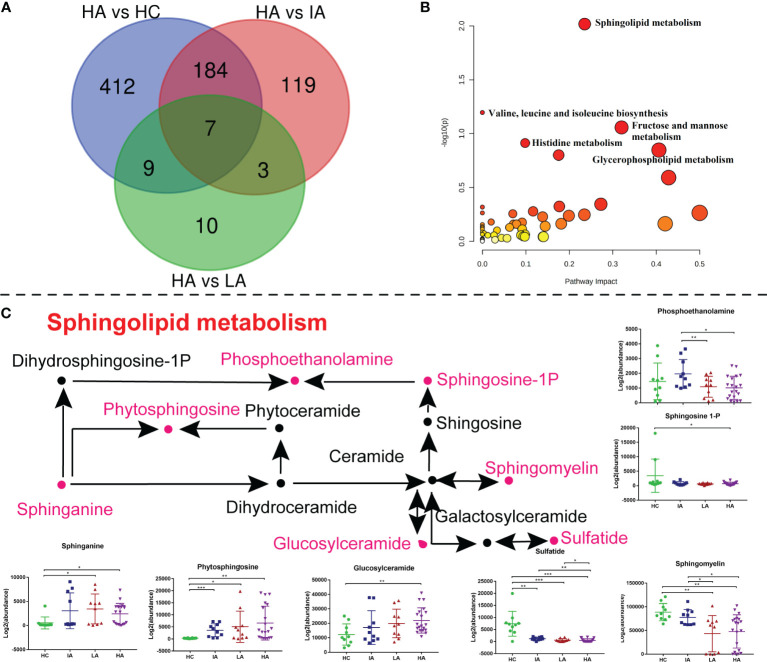
Sphingolipid Metabolism Perturbations in High Activity Stage of SLE. **(A)** Venn diagram showing the number of differentially expressed metabolites in HA compared with HC, IA, LA groups, respectively. **(B)** KEGG analysis of differentially expressed metabolites in HAs compared with others. **(C)** A summary of sphingolipid metabolism pathways. Box plots show expression level changes of selected regulated metabolites in sphingolipid metabolism pathways across four disease severities. P values were calculated using Student’s t test and significant P values are shown in the boxplot. * means P value < 0.05, ** means P value < 0.01, and *** means P value < 0.001.

As mentioned above, the metabolomics data revealed a significant impact on sphingolipid metabolism (**Figure 3B**). To determine whether this pathway is also involved in high disease activity, differentially expressed metabolites between HAs and other groups were identified (**Figure 7A**). Of note, sphingolipid metabolism was the top pathway affected in the high activity group from our metabolomics analysis (**Figure 7B** and **Supplementary Data 3.2**). As such, further analysis of this pathway highlighted significant increases in sphinganine, glucosylceramide, and phytosphingosine levels (**Figure 7C**). In contrast, decreases in sulfatide, sphingomyelin (SM), and sphingosine-1P suggested hyperactivation of the ceramide pathway in activity stage of SLE. Among them, glucosylceramide and SM levels were also associated with SLE disease score (**Supplementary Figure 6B**). Consistently, sphinganine and SM were reported to be changed in SLEs and related to complications, thus supporting our data (20). These data show the potential value of assessing sphingolipid changes in SLEs, especially in the HA group, and supports the use of sphingolipidomics as a tool to pinpoint a biomarker for early identification (12).

## Discussion

To the best of our knowledge, this is the first integrated multi-omic analysis combining proteomic, metabolomic and lipidomic data obtained from SLE patients with different disease activity. Previous studies have observed abnormal alterations in many mRNAs, proteins and metabolites in SLE patients. For example, elevated expression of CTRP3 and TRYP2, which might participate in the pathogenesis, were observed in plasma from neuropsychiatric SLE (21). Additionally, serum properdin, collectin-11 and thrombospondin-4 levels were able to monitoring the diseases activity of SLE patients (6). These studies help to elucidate the pathogenesis of SLE; however, a common limitation of these studies is that they were performed using single omics technology. Recently, many researchers have applied multiple omics technologies and showed that this approach was able to provide a greater detailed view of the underlying mechanism of disease than when used individually (22, 23). However, to date there have been limited studies which have focused on a combined analysis of proteomics and metabolomics in SLE.

Multi-omics research from proteomics to metabolomics allows researchers to establish a systematic analysis to clarify the complete molecular pathology of SLE. In this study, we performed proteomics, metabolomics and lipidomics simultaneously in patients with different SLE activity stage and HCs. Through multi-omics analysis, levels of immune-related and inflammation-related proteins were found to be elevated in SLE patients compared to HCs. Unexpectedly, compared with LA patients, HA patients exhibited a discordance, with activation of inflammation but low IgG and immunonoglobulins secretion. Additionally, the number of B cells were also decreased in HAs and this was correlated with the levels of immunoglobulins. This indicates that patients in the high activity group might have a certain degree of immune damage. It is known that the innate immune system is activated by TLRs, which leads to downstream activation of MAP-kinases and ERK1/2 signaling pathways, which serve as transcription factors for the production of pro-inflammatory mediators (24). In this study, we observed several inflammation-related proteins in the MAP-kinases and ERK1/2 signaling pathways were significantly altered in SLE patients, consistent with previous studies (25). Acute inflammation proteins were also increased, implying that inflammation plays a key role in the pathogenesis of SLE. Moreover, cytokines such as TNF-α, IL-2R, IL-6, IL-18 were also increased in HCs and showed similar trends with inflammation-related proteins. These results suggested that the pathogenesis of SLE is accompanied by inflammatory activation and immune dysfunction, which play an essential role in the activity of SLE.

Together with the adaptive immune response, the complement system also plays an essential role in immunity. Complement activation is associated with systemic lupus erythematosus with expression of complement proteins decreased and complement deposition found in affected tissues (26). However, the coordinated changes in complements have not been analyzed as usually each molecule is analyzed independently using specific immunoassays. In this study, both proteomics and clinical indexes indicated that complement components, such as C1QC, C1R, C4A, C4B and others were significantly decreased in SLE and associated with disease activity. This revealed the importance of the classical pathway for lupus pathogenesis. The role of the complement system in SLE is complex. Complement activation can help aggravate SLE-associated inflammation while complement deficiency is also dangerous as it can exacerbate SLE activity (27). It has been reported that the complement component of the classical pathway, such as C1Q, C1R, C4 and C2, is genetically defective and involved in the progression of SLE (27, 28). Additionally, functional defects of C1Q can lead to a decrease in its ability to detect apoptotic cells, which further promotes progression of SLE (29). Moreover, low copy numbers of the two C4 genes, C4A and C4B, is also associated with increased risk of SLE (30), thus supporting our data. Together, these evidence show that (i) SLE is a multi-systemic and complex autoimmune disease, characterized by excessive inflammation (31); (ii) immunoglobulins facilitate inflammation activation in SLE; and (iii) The degree of complement cleavage is determined by the levels of inflammation response (32). These results suggest that the adaptive immune system, complement system and inflammation interacted with each other, influenced each other and are jointly involved in SLE disease activation.

Dyslipidemia is a common feature in patients with SLE, and includes elevated LDL, HDL and APOB (19). However, beyond these findings, very little information is available to assess whether defects in lipid metabolism are present in SLE. In this study, in addition to APOB, levels of APOC, APOD, APOE, and APOL1 were also significantly increased in SLE, especially in HA patients. This suggests that abnormal lipids metabolism not only occurs in SLE pathogenesis but is also associated with SLE disease activity. In addition, using metabolomics, we found that the lipidomics characteristics of SLE patients were significantly different from HCs. Importantly, sphingolipid metabolism was identified as the top pathway associated with high activity in SLE, consistent with previous research (12). Studies have shown that dyslipidemia may elevate the cardiovascular risk of SLEs (33, 34). Since atherosclerosis is induced by an imbalance in lipid metabolism, it is possible that the hyperlipidemic environment *in vivo* induced by dysregulated lipid metabolism is involved in the pathogenesis of SLE (35). Future research should investigate the mechanisms by which specific lipid contents stimulate autoimmune and inflammatory responses to promote the occurrence and development of SLE.

It should be noted that this was a single-center study with a relatively small sample size. Therefore, future large-sized cohort studies are necessary to confirm the results in this research. Additionally, some of the enrolled SLE patients had received treatment for more than a month. Thus, due to the cross-sectional nature of this study, correlation does not necessarily imply causation, especially when these factors include potential common dependent variables.

In summary, our study presented a multi-omics landscape of blood samples within a cohort of SLE patients with different disease activity, from non-activity to high activity. Overall, this study provides novel insights into SLE as well as valuable clues for deciphering the pathogenesis of SLE and its underlying mechanisms.

## Data Availability Statement

The original contributions presented in the study are included in the article/**Supplementary Material**. Further inquiries can be directed to the corresponding authors.

## Author Contributions

YW and JQL designed the study. JT Supervised the study. XH, NJ, JZ, JF, FX, CL, SL, GS, JH, MK, DZ, YX, XC, and JML contributed to samples, materials and reagents. YW, JQL, and LL analyzed the data. JQL conducted the software. YW and JQL drafted the manuscript. LL revised the manuscript. All authors contributed to the article and approved the submitted version.

## Funding

This work was supported by grants from Public service development and reform pilot project of Beijing Medical Research Institute (BMR2019-11), National natural science foundation of China (81970900) and Beijing Social Science Foundation Project (19GLB033).

## Conflict of Interest

The authors declare that the research was conducted in the absence of any commercial or financial relationships that could be construed as a potential conflict of interest.

## Publisher’s Note

All claims expressed in this article are solely those of the authors and do not necessarily represent those of their affiliated organizations, or those of the publisher, the editors and the reviewers. Any product that may be evaluated in this article, or claim that may be made by its manufacturer, is not guaranteed or endorsed by the publisher.

## Supplementary Material

Supplementary Figure 1 Quality Control and Differentially Expressed Proteins in Different Groups, Related to **Figure 2**. **(A)** The distribution of the numbers of quantified peptides and proteins in the 50 plasma samples **(B)** PLS-DA was used to compare the proteomes of the different groups. **(C)** Volcano plots comparing protein expression between groups. Proteins with |FC| >1.5 and P value < 0.05 were considered to be significantly differentially expressed.

Supplementary Figure 2 Expression of Representative Proteins in the Decreasing Clusters (C4, C5 and C6), Related to **Figure 2**. **(A)** Levels of tropomyosin (TPM) among HCs, IAs, LAs and HAs. **(B)** Levels of collagen proteins among HCs, IAs, LAs and HAs. **(C)** Levels of integrin proteins among HCs, IAs, LAs and HAs.

Supplementary Figure 3 Quality Control and Differentially Expressed Metabolites in Different Groups, Related to **Figure 3**. **(A)** PLS-DA score plot used to compare the metabolites of different groups and demonstrates the reliability of the analytical method used on QC samples (n = 4). **(B)** RSD% of all detected variables. Number (%): percentage of the number of variables. Sum of intensity (%): percentage of the total response of the variables. **(C)** Volcano plots comparing metabolite expression between groups. Metabolites with |FC| >1.5 and P value < 0.05 were considered to be significantly differentially expressed.

Supplementary Figure 4 Quality Control and Differentially Expressed lipids in Different Groups, Related to **Figure 3**. **(A)** PLS-DA score plot used to compare the lipids of different groups and demonstrates the reliability of the analytical method used on QC samples (n = 4). **(B)** RSD% of all detected variables. Number (%): percentage of the number of variables. Sum of intensity (%): percentage of the total response of the variables. **(C)** Volcano plots comparing lipid expression between groups. Lipids with |FC| >1.5 and P value < 0.05 were considered to be significantly differentially expressed.

Supplementary Figure 5 Correlation Analysis of Omics-data and Clinical Index. **(A)** Correlation analysis of the immunoglobulin proteins versus B cell numbers and antibody levels. **(B)** Spearman correlation heatmap between levels of differentially expressed apolipoproteins and different lipids. The result was obtained using Pearson correlation coefficient analysis.

Supplementary Figure 6 Correlation of Metabolites and Lipids with SLE Disease Score. **(A)** Metabolite levels were correlated to SLE disease score. **(B)** Lipid levels were correlated to SLE disease score. *x* axis indicates Spearman’s correlation coefficients, and the *y* axis indicates the significance of the correlation (–log_10_ of P values for each correlate).
