# Supplementary figures and images for: Multi-Platform Omics Analysis Reveals Molecular Signatures for Pathogenesis and Activity of Systemic Lupus Erythematosus
Source: Front Immunol. 2022 Apr 19;13:833699. doi: 10.3389/fimmu.2022.833699 (PMC9063006; doi:10.3389/fimmu.2022.833699)

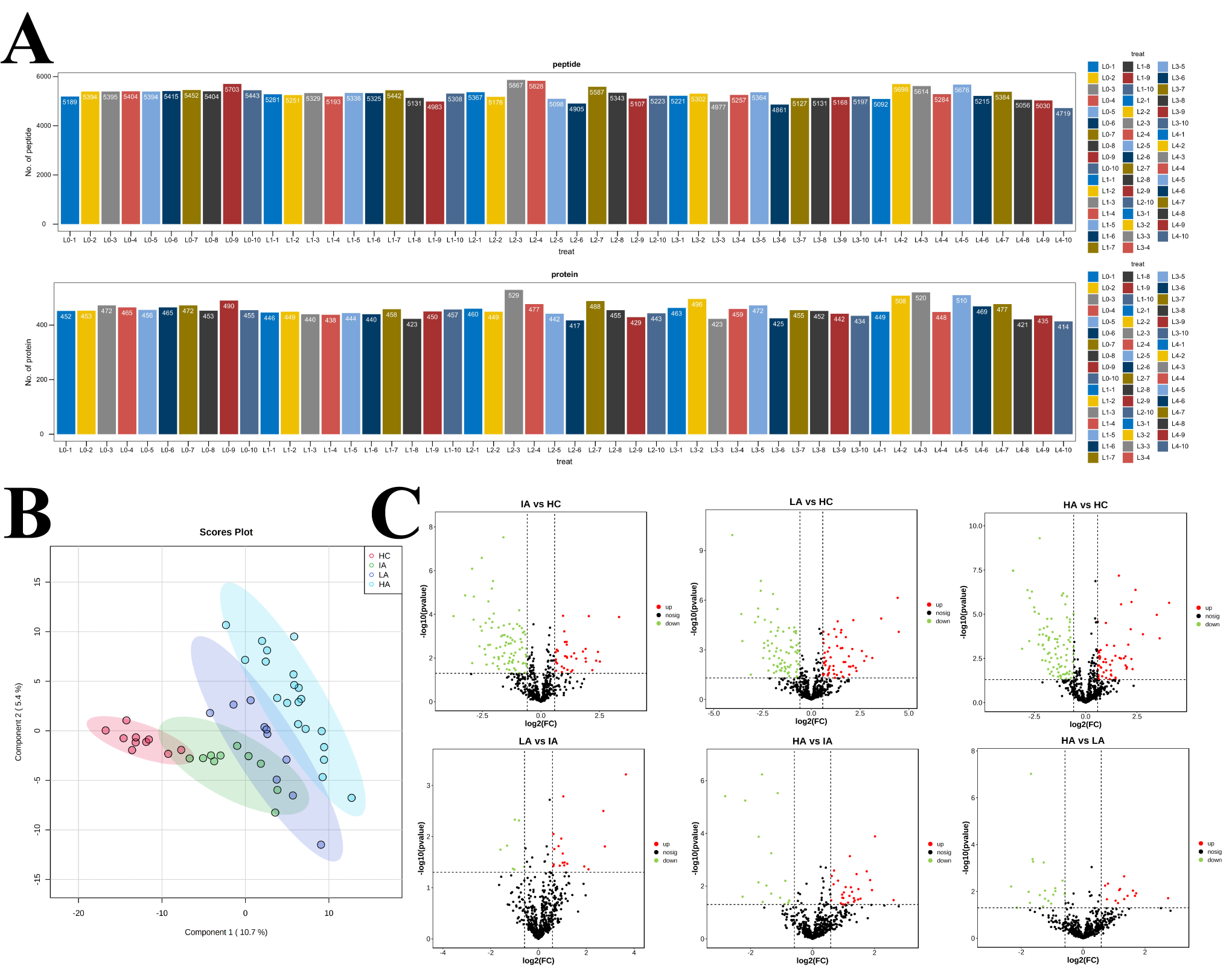

Supplement: Supplementary Figure 1 — Quality Control and Differentially Expressed Proteins in Different Groups, Related to Figure 2 . (A) The distribution of the numbers of quantified peptides and proteins in the 50 plasma samples (B) PLS-DA was used to compare the proteomes of the different groups. (C) Volcano plots comparing protein expression between groups. Proteins with |FC| >1.5 and P value < 0.05 were considered to be significantly differentially expressed. [file Image_1.tif]

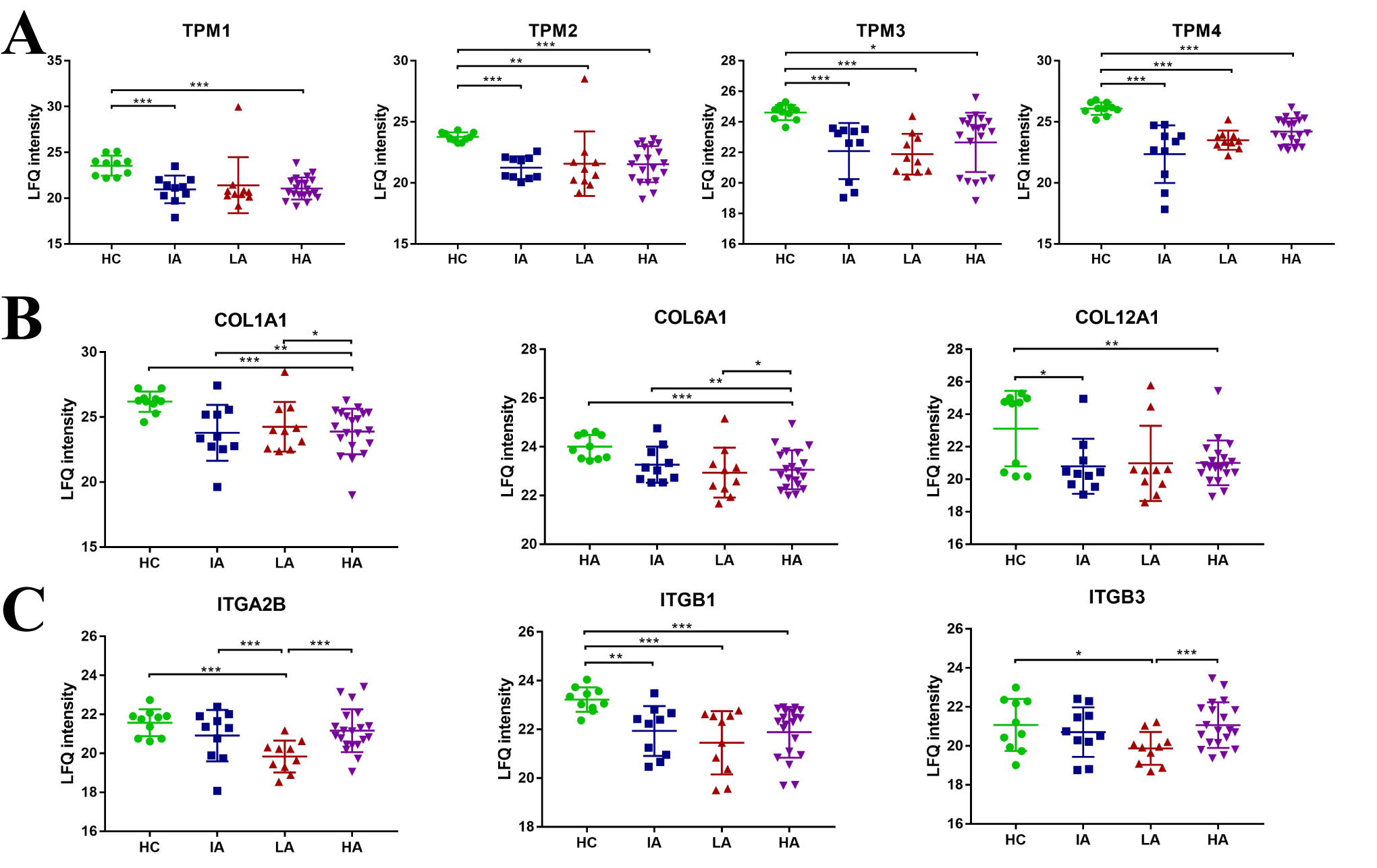

Supplement: Supplementary Figure 2 — Expression of Representative Proteins in the Decreasing Clusters (C4, C5 and C6), Related to Figure 2 . (A) Levels of tropomyosin (TPM) among HCs, IAs, LAs and HAs. (B) Levels of collagen proteins among HCs, IAs, LAs and HAs. (C) Levels of integrin proteins among HCs, IAs, LAs and HAs. [file Image_2.tif]

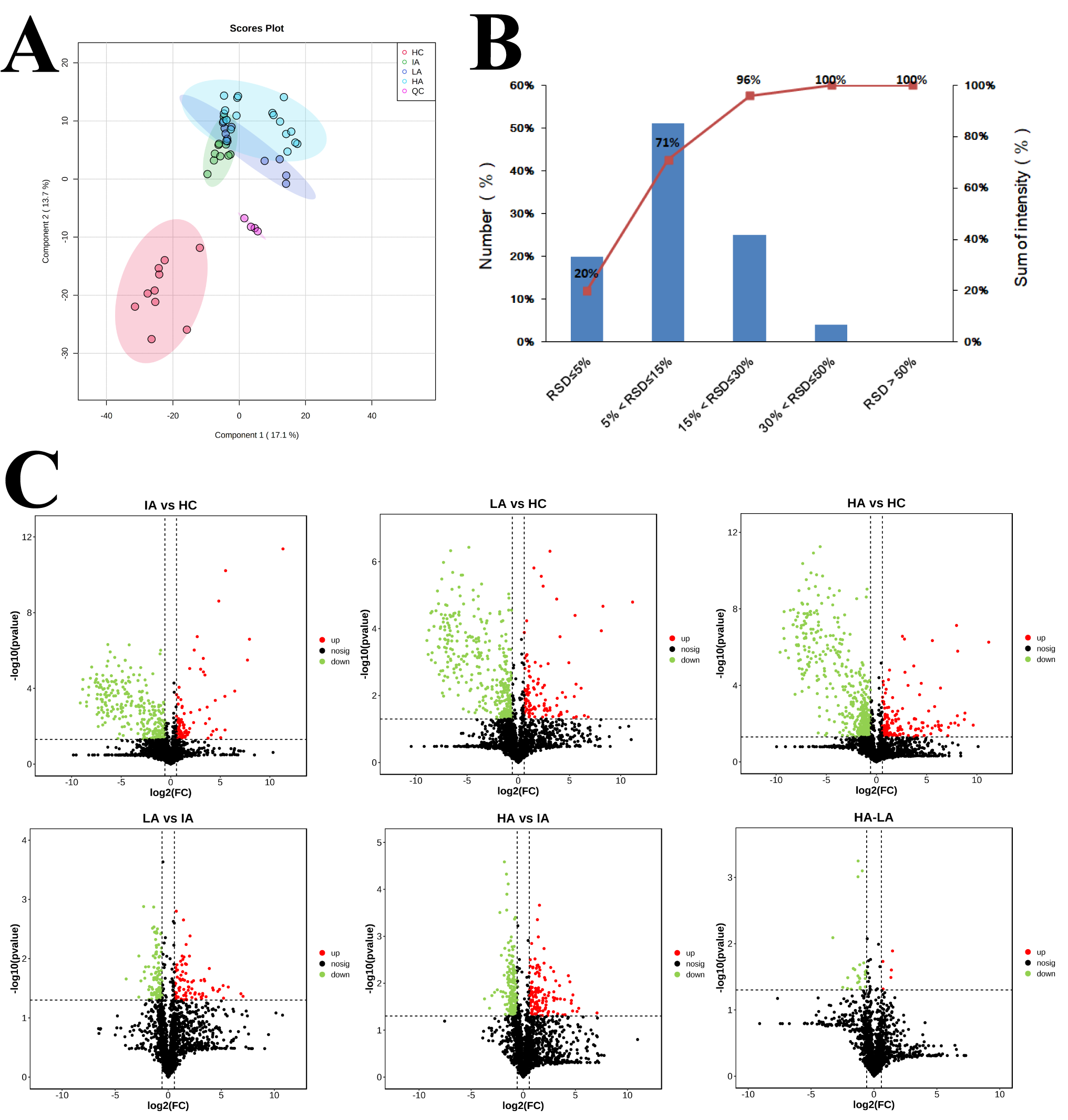

Supplement: Supplementary Figure 3 — Quality Control and Differentially Expressed Metabolites in Different Groups, Related to Figure 3 . (A) PLS-DA score plot used to compare the metabolites of different groups and demonstrates the reliability of the analytical method used on QC samples (n = 4). (B) RSD% of all detected variables. Number (%): percentage of the number of variables. Sum of intensity (%): percentage of the total response of the variables. (C) Volcano plots comparing metabolite expression between groups. Metabolites with |FC| >1.5 and P value < 0.05 were considered to be significantly differentially expressed. [file Image_3.tif]

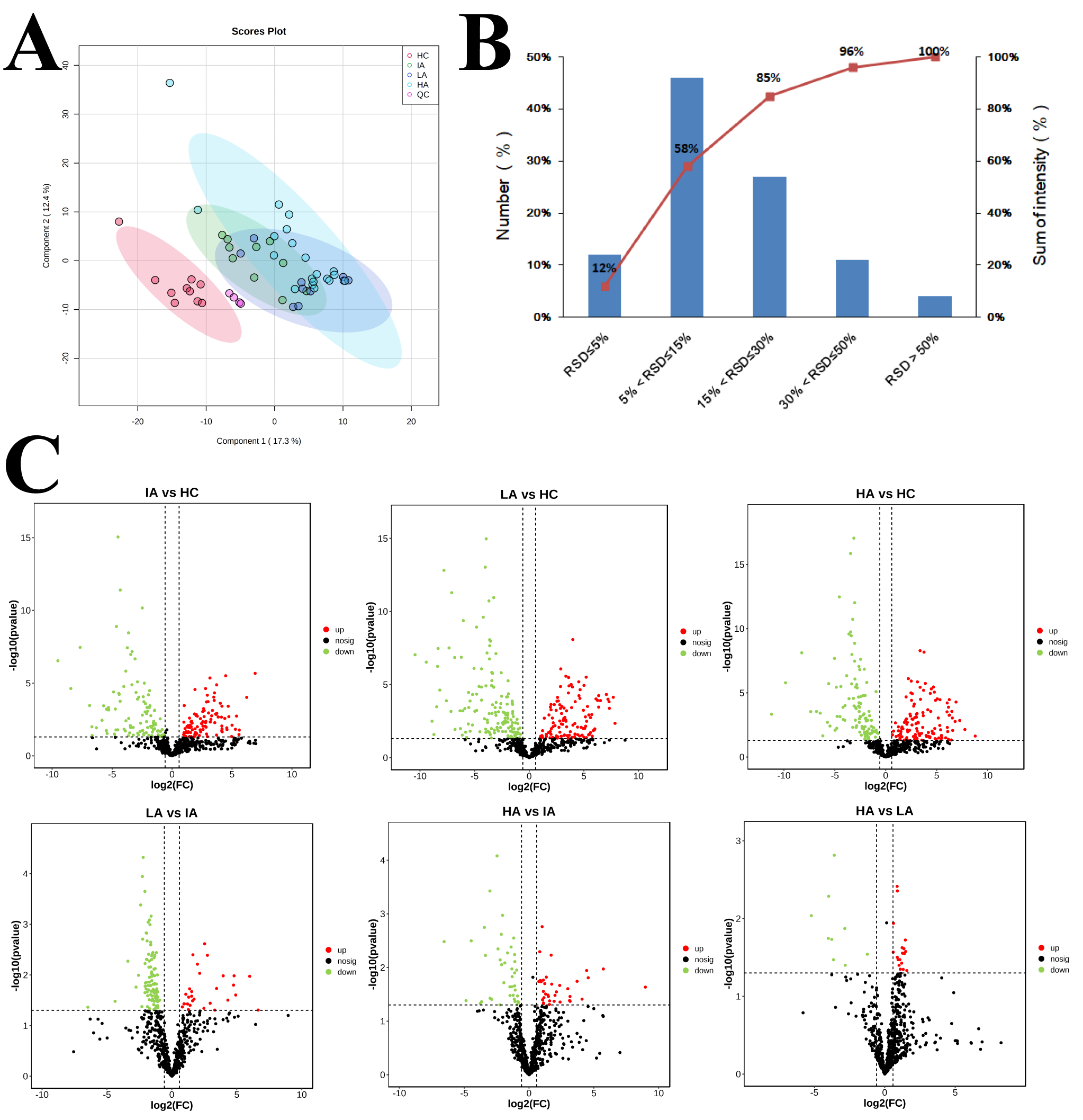

Supplement: Supplementary Figure 4 — Quality Control and Differentially Expressed lipids in Different Groups, Related to Figure 3 . (A) PLS-DA score plot used to compare the lipids of different groups and demonstrates the reliability of the analytical method used on QC samples (n = 4). (B) RSD% of all detected variables. Number (%): percentage of the number of variables. Sum of intensity (%): percentage of the total response of the variables. (C) Volcano plots comparing lipid expression between groups. Lipids with |FC| >1.5 and P value < 0.05 were considered to be significantly differentially expressed. [file Image_4.tif]

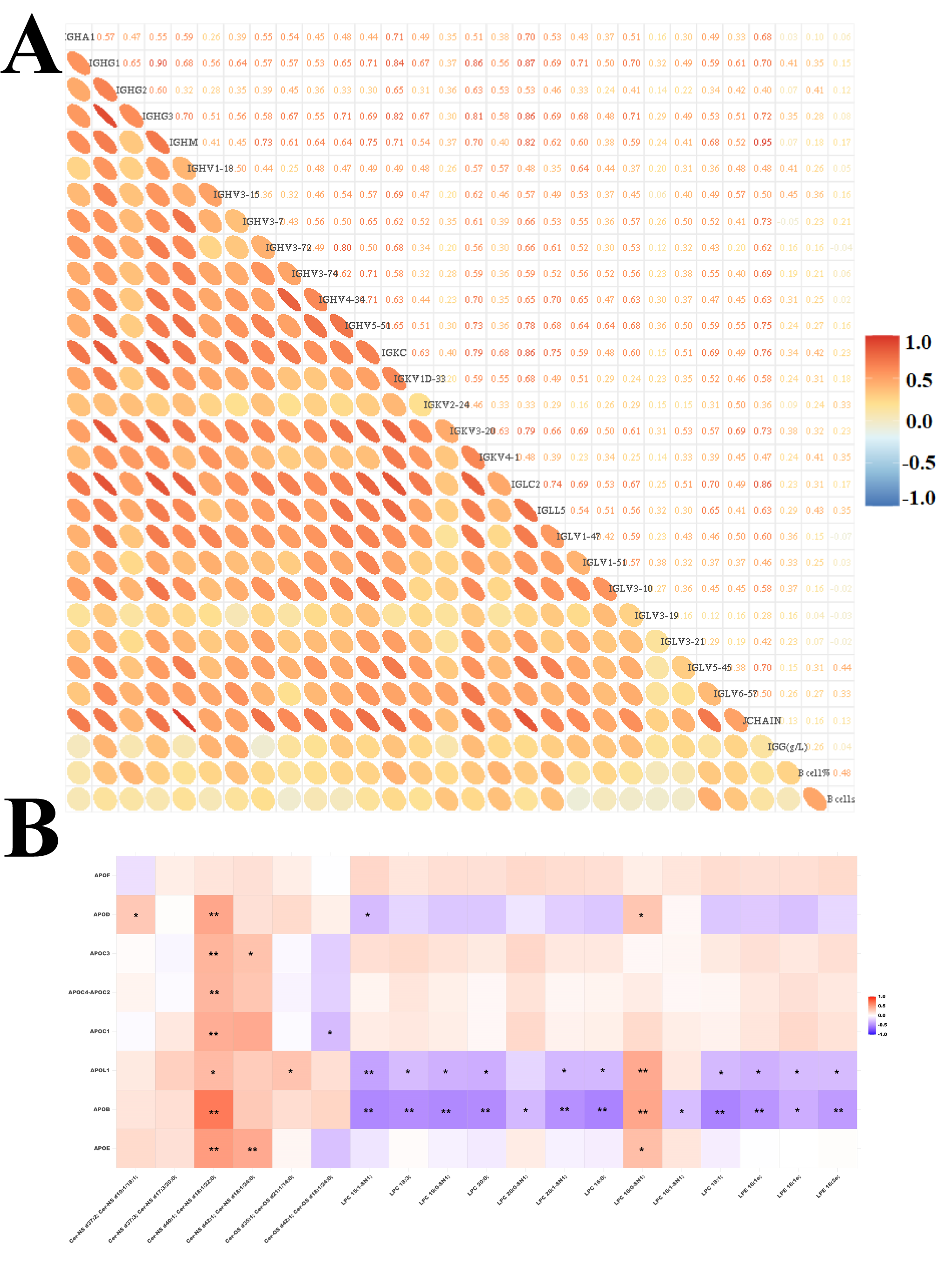

Supplement: Supplementary Figure 5 — Correlation Analysis of Omics-data and Clinical Index. (A) Correlation analysis of the immunoglobulin proteins versus B cell numbers and antibody levels. (B) Spearman correlation heatmap between levels of differentially expressed apolipoproteins and different lipids. The result was obtained using Pearson correlation coefficient analysis. [file Image_5.tif]

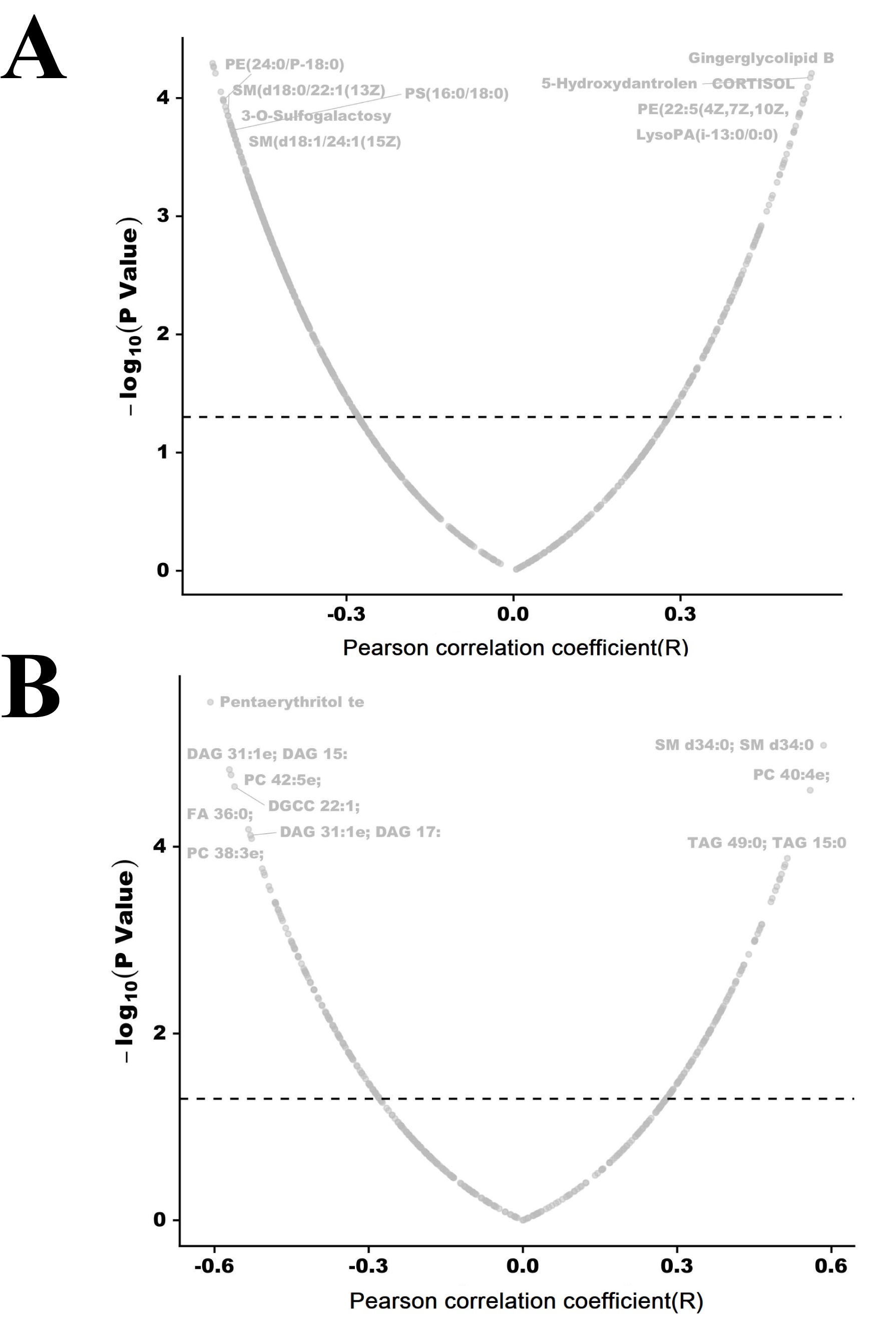

Supplement: Supplementary Figure 6 — Correlation of Metabolites and Lipids with SLE Disease Score. (A) Metabolite levels were correlated to SLE disease score. (B) Lipid levels were correlated to SLE disease score. x axis indicates Spearman’s correlation coefficients, and the y axis indicates the significance of the correlation (–log10 of P values for each correlate). [file Image_6.tif]
